# Molecular detection of *Setaria tundra* (Issaitshikoff & Rajewskaya, 1928) and *Setaria cervi* (Rudolphi, 1819) in red deer in Slovakia

**DOI:** 10.1007/s11259-024-10394-0

**Published:** 2024-06-14

**Authors:** Jozef Lazár, Júlia Šmigová, Ľubomír Šmiga, Federica Berrilli, Peter Lazár, Ján Čurlík, Ingrid Papajová

**Affiliations:** 1 Department of Breeding and Diseases of Game, Fish and Bees, Ecology and Cynology, University of Veterinary Medicine and Pharmacy in Košice, Komenského 73, Košice, 041 81 Slovak Republic; 2 Institute of Parasitology, Slovak Academy of Sciences, Hlinkova 3, Košice, 040 01 Slovak Republic; 3 Department of Clinical Sciences and Translational Medicine, Faculty of Medicine, University of Rome “Tor Vergata”, Via Montpellier 1, Rome, 001 33 Italy

**Keywords:** Cervid parasites, Filaroid nematode, Vector-borne disease

## Abstract

Filaroid nematodes *Setaria tundra* (Issaitshikoff & Rajewskaya, 1928) and *Setaria cervi* (Rudolphi, 1819) are internal parasites from family Onchocercidae with occurrence in the northern hemisphere. They have a considerably wide range of final host, including many species of family Cervidae. Intermediate hosts and vectors at the same time, are represented by the several mosquito species, mostly of genus *Aedes.* Infection of *Setaria* is relatively harmless and especially in wild cervids usually pass unnoticed. Although in some cases it can induce peritonitis which might be a life threatening condition.

This study was determined to reveal the presence of helminths *Setaria tundra* and *Setaria cervi* in red deer (*Cervus elaphus*) in Slovakia. The parasites were identified morphologically and genetically, based on the sequences of a fragment of the mitochondrial cytochrome c oxidase subunit 1 (*cox*1) gene. For this purpose we used partial results of our longer parasitological monitoring realized in one particular hunting area located in eastern Slovakia, near the city of Košice. A total of 60 red deer individuals were tested, of which one was found to be infected with *Setaria tundra* (prevalence of 1.7%) and four were detected to be infected with *Setaria cervi* (prevalence 6.7%). The intensity of infection was very low, only one specimen of *Setaria* spp. in each positive animal.

## Introduction

Filaroid nematodes *Setaria tundra* (Issaitshikoff & Rajewskaya, 1928) and *Setaria cervi* (Rudolphi, 1819) are internal parasites from family Onchocercidae. These parasites have an indirect life cycle that includes mosquitoes as an intermediate host and final hosts are represented by animals of the *Cervidae* and *Bovidae* families (Taylor et al. 2007; Laaksonen and Paulsen 2015). Mosquitoes play an important role not only as intermediate hosts but also as a vector of this parasitic infection. Most competent species for transmission *S. tundra* are supposed to be mosquitoes of genus *Aedes*, especially *Ae. vexans*. Besides the genus *Aedes*, suitable vectors are also mosquito species of genera *Anopheles*, *Ochlerotatus*, *Culex* and *Coquillettidia* (Oloś et al. 2021). Demiaszkiewicz et al. 2015 in Poland found that the intermediate host of *S. cervi* is *Haematobia stimulans*.

Both species are long, thread-like nematodes, milk-white in color (Nikander et al. 2007). Presently, the genus *Setaria*, Viborg, 1795, contains 46 species that parasitize in the peritoneal cavity of Artiodactyla, Perissodactyla and Hyracoidea. The adult parasites dwell in the peritoneal cavity, where the females produce first-stage larvae (*microfilariae*) that enter the host’s bloodstream. First-stage larvae are then uptake by mosquitoes (intermediate host/vector) during their blood feeding, so parasites can subsequently reach the infectious stage (Laaksonen and Paulsen 2015).

Infection of *Setaria* spp. is usually harmless and only occasionally inducing fibrinous peritonitis associated with poor body condition, especially in young animals (Laaksonen et al. 2007), this phenomenon was recorded in domestic and semi-domestic reindeer (*Rangifer tarrandus*) in Finland, which caused massive economic losses. The outbreak of serofibrinous peritonitis was noticed by meat-inspecting veterinarians in slaughterhouses (Laaksonen et al. 2007; Enemark et al. 2017).

There have been a multiple reports of *S. tundra* occurrence in roe deer (*Capreolus capreolu*s) in many countries across the Europe, most importantly in countries neighboring with Slovakia such as Austria (Kutzer and Hinaidy 1969), Hungary (Kemenesi et al. 2015) and Poland (Kowal et al. 2013; Tomczuk et al. 2017) moreover in elk (*Alces alces*) in Poland (Demiaszkiewicz et al. 2015). But there is not as many reports of *S.cervi* in red deer (Alasaad et al. 2012; Oloś et al. 2019; Lanková et al. 2021).

Therefore the aim of this study was to confirm the presence helminths of genus *Setaria* Viborg, 1795 (*Filarioidea*, Onchocercidae) in red deer (*Cervus elaphus*) in Slovakia by using molecular methods, which could be considered as a follow-up work to previous study about *S. tundra* in roe deer, done by Čurlík et al. (2023).

## Materials and methods

For the purpose of this study we were focused on red deer (*Cervus elaphus*) Linnaeus, 1758. Study was conducted in the one particular hunting area which is located in a range of mountains in eastern Slovakia (in Slovak: Slanské vrchy), approximately 30 km far from the city of Košice. The executive organ of hunting management in mentioned hunting area is University of Veterinary Medicine and Pharmacy in Košice. The size of hunting area is 2 300 hectares of land, mostly covered by deciduous forest with a few meadows located in lower parts. This region has moderate climate and altitude in hunting area ranging from (400 m asl) in lower parts to highest point Makovica Hill (981 m asl).

All animals in the presented paper have been killed during three consecutive hunting seasons (2020/2021, 2021/2022 and 2022/2023). In total, we managed to perform a post mortem examination in 60 red deer individuals (17 males, 25 females and 18 fawns). According to valid Slovak hunting regulations, is the date of hunt for males and fawns from 1 August to 15 January and females from 1 August to 31 December, exceptions may occur if there is a valid reason such as an unfilled annual quota for young males or fawns. The positive animals were culled– fawns in February 2022, first young male in January 2021 and second young male in March 2022, and old male in February 2023.

The *Setaria* nematodes were found either during the procedure of evisceration in the field conditions soon after the animal has been killed or during examination of internal organs at the necropsy room. The *Setaria* nematodes which were found within the infected host animals were located on the surface of the intestines and rumen. Other parts of the animal (spinal cord, brain) were not dissected. If it was possible, we additionally performed basic parasitological examination of internal organs such as lungs, liver and digestive tract (abomasum, small intestine, and large intestine).

Collected nematodes *Setaria* spp. were rinsed in saline solution, and their morphological structures were analyzed under the light microscope according to recent morphological keys (Nikander et al. 2007; Oloś et al. 2019; Lanková et al. 2021). Helminths were subsequently stored in 96% ethyl alcohol for further molecular analyses. Species diagnostics were confirmed by using molecular methods.

### Extraction, amplification of DNA and sequence analysis

Genomic DNA was extracted from adult nematode identified morphologically as *S. tundra*, using the QIAamp DNA Mini Kit (Qiagen, Germany) according to the manufacturer’s instructions. Amplification of a partial sequence of the mitochondrial cytochrome oxidase subunit I (COI) gene was performed using the forward primer cox1int F (5’-TGATTGGTGGTTTTGGTAA-3’) and reverse primer cox1int R (5’-ATAAGTACGAGTATCAATATC-3’) (Casiraghi et al. 2001). The resulting fragment was approximately 690 bp in length. For PCR reaction to amplify the COI fragment was used total volume of 25 µl consisted 5 µl of extracted parasitic DNA, 1X DreamTaq Green Buffer, 1 mM MgCl2, 200 µM of each dNTP, 500 nM of each of the primers, 2.5 U of HotStarTaq DNA Polymerase (Qiagen, Germany).

PCR was performed as follows: initial denaturation was performed at 95 °C for 2 min, followed by 35 cycles of denaturation at 94 °C for 1 min, annealing at 52 °C for 1 min and elongation at 72 °C. Final extension was performed at 72 °C for 5 min in the MyCycler™ Thermal Cycler System (Bio-Rad Laboratories, Berkeley, CA, USA). Amplicons were separated on a 1.5% agarose gel stained with Goodview Nucleic Acid Stain (SBS Genetech Co., China) and TAE buffer (40 mM Tris, pH 7.8, 20 mM acetic acid, 2 mM EDTA).

Positive PCR products were purified using the ISOLATE II PCR and Gel Kit (Bioline, UK). DNA sequencing was performed on an automated DNA sequencer ABI prism 3700 at the University of Veterinary Medicine and Pharmacy, Košice, Slovakia. The obtained DNA sequences were compared with the reference sequences of in the GenBank database by the nucleotide BLASTn (https://blast.ncbi.nlm.nih.gov) program and were grouped by similarity and aligned using the MEGA 11 (Tamura et al. 2021). The Tamura-Nei model was chosen as the most appropriate model for the data analysed. All positions containing gaps and missing data were eliminated. *Thelazia callipaeda* was chosen as an outgroup. Bootstrap analysis was performed with 1,000 replicates to test robustness of the phylogeny.

## Results

By the available descriptions of morphological features in the literature, all nematodes found in the abdominal cavity were classified as adults of genus *Setaria* spp. This species diagnostic was based on specific localization in host (peritoneal cavity) and on the structure of the peribuccal crown on the cephalic part of the parasite as well as on the morphology of their caudal end (Nikander et al. 2007; Čurlík et al. 2019; Oloś et al. 2019; Lanková et al. 2021; Oehm et al. 2022).

Individuals of *Setaria* spp. were found in 5 out of 60 (prevalence 8.3%) examined red deer (Cervidae, *Cervus elaphus*) individuals (in 2 fawns - females; in 2 males of age 2–3 years and in 1 male of age 7–8 years). All five positive animals were in adequate body condition except for one young male (killed on 4. February 2023 with bodyweight only 60 kg) which was in poor body condition. No apparent clinical signs or pathological lesions associated with *Setaria* infection were observed before animals were killed or during post mortem examination. The intensity of infection was very low, only one specimen of *Setaria* spp. in each positive animal. We did not observe any difference in prevalence between individual years.

**Fig. 1:**
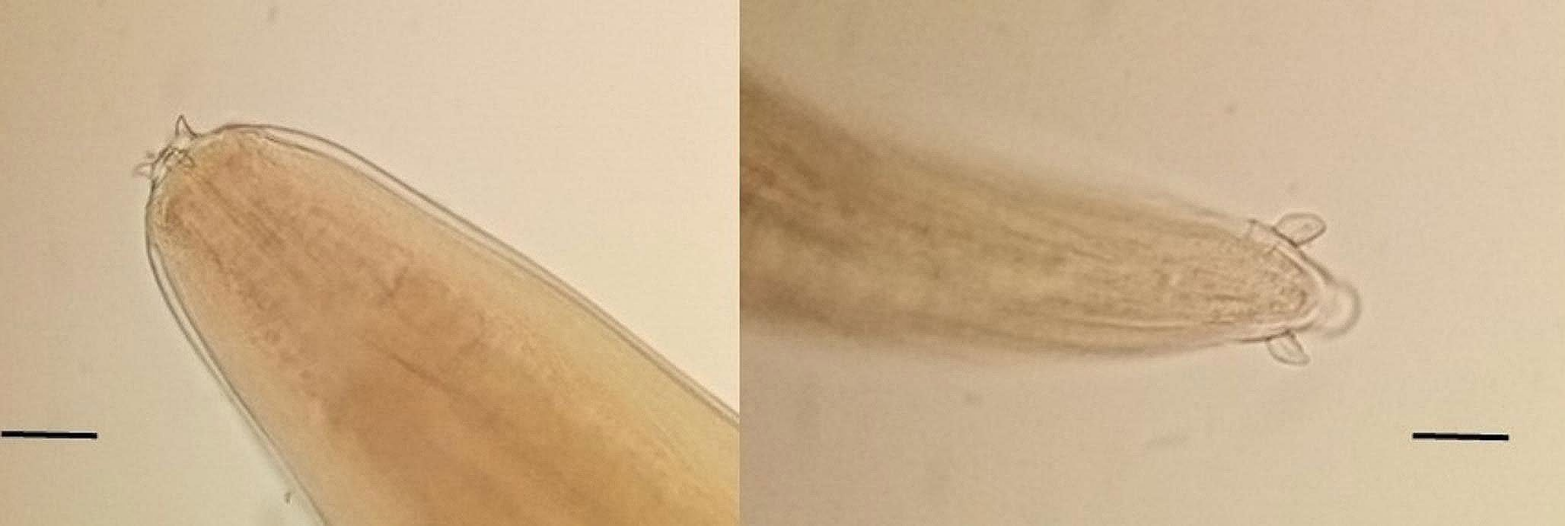
The peribuccal crown on the rounded cephalic body end (left side; the scale bar 100 μm) and lateral appendages on caudal end (right side; the scale bar 50 μm) of *Setaria cervi* female

**Fig. 2:**
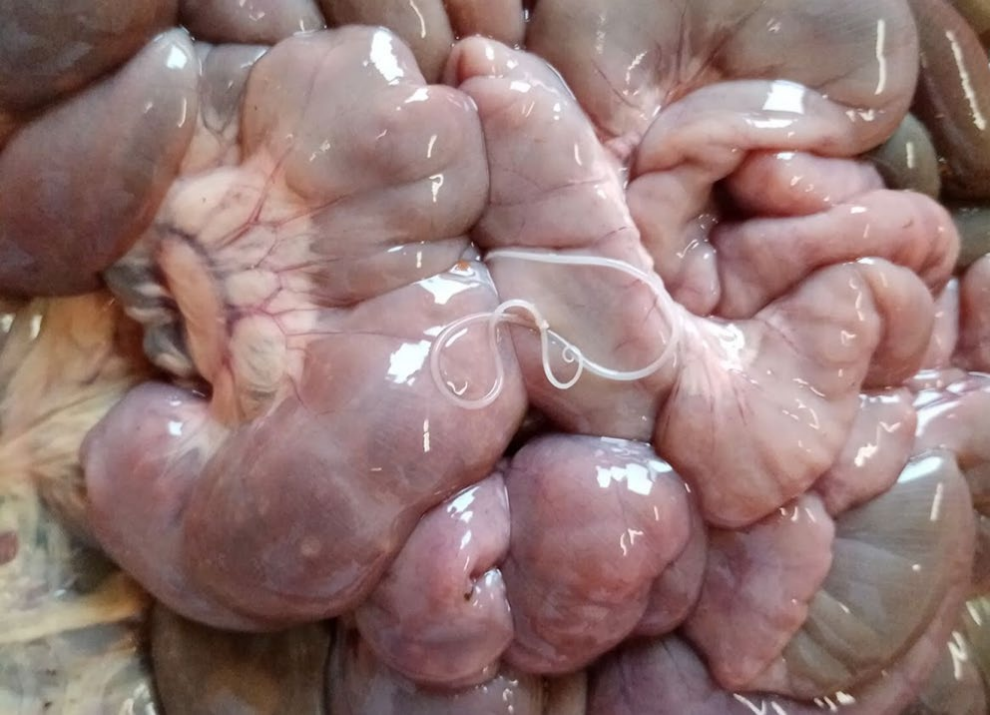
The nematode *Setaria cervi*, female localised on the surface of intestine in fawn of red deer

**Fig. 3:**
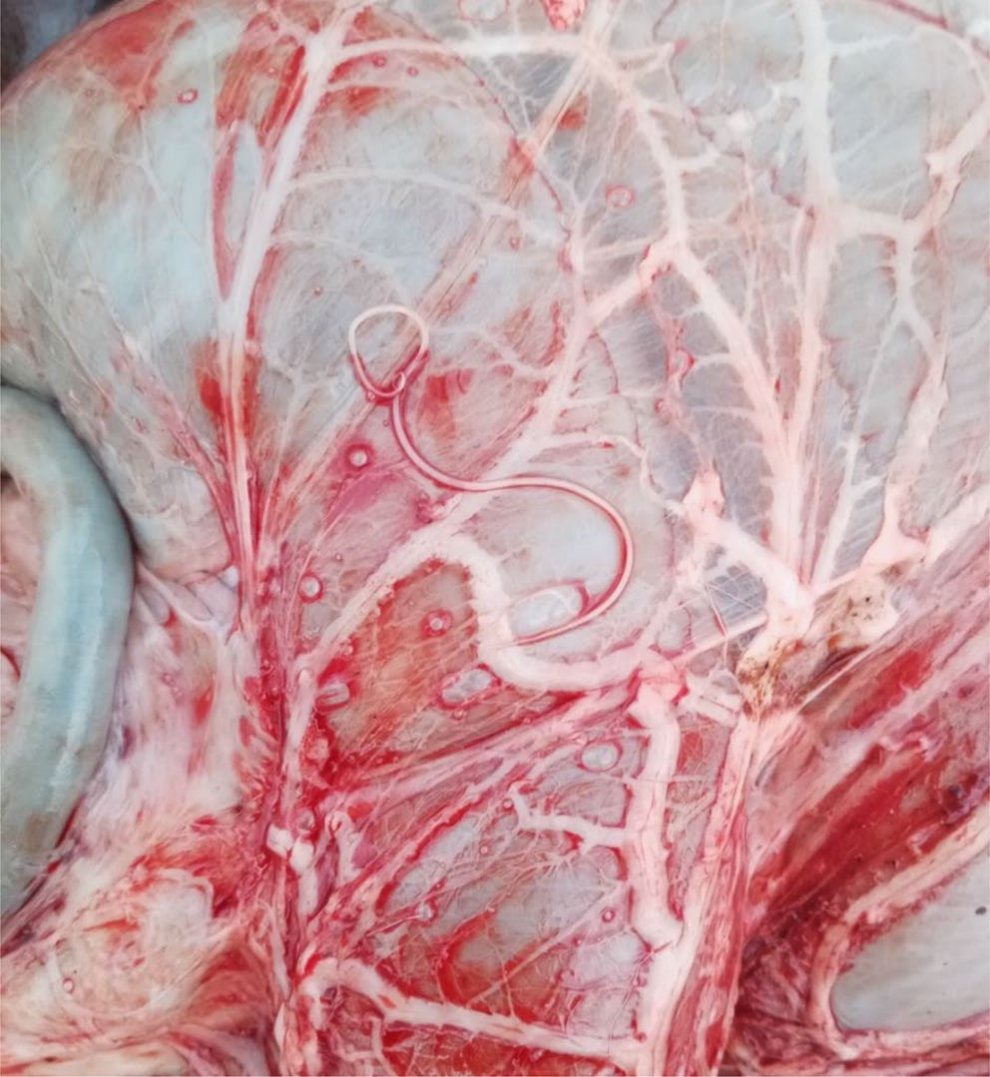
The nematode *Setaria cervi*, female localised on the surface of rumen in young male of red deer

Based on morphology (Fig. 1.) and molecular diagnostic, one of the five helminths was identify as *S. tundra* (in the oldest male) and remaining four of the *Setaria helminths* were identify as a *S. cervi.* All collected nematodes were adult females (*S. tundra* length– 51 mm, *S. cervi* average length 121 mm) which were localised on surface of intestine (Fig. 2) or rumen (Fig. 3). In addition, in 4/5 individuals with *Setaria* infection there were also present infection of gastrointestinal nematodes, in 2/5 there were present infections of pulmonary nematodes.

Genomic DNA was successfully extracted from 5 adult individuals of nematodes species *Setaria*. Amplification of COI gene regions was successful in five isolates of the parasitic DNA. The final amplified fragment was approximately 690 bp. Morphological analyses supported by molecular analyses are the best approach to identify *Setaria* nematodes. Both analyses were applied to all nematode individuals in our study.

Accession numbers of the sequences obtained were stored in GenBank. Individual of *S. tundra* was deposited under accession number: PP196346 and *S. cervi* under accession numbers: PP198315; PP198852; PP198854; PP208633.

**Fig. 4:**
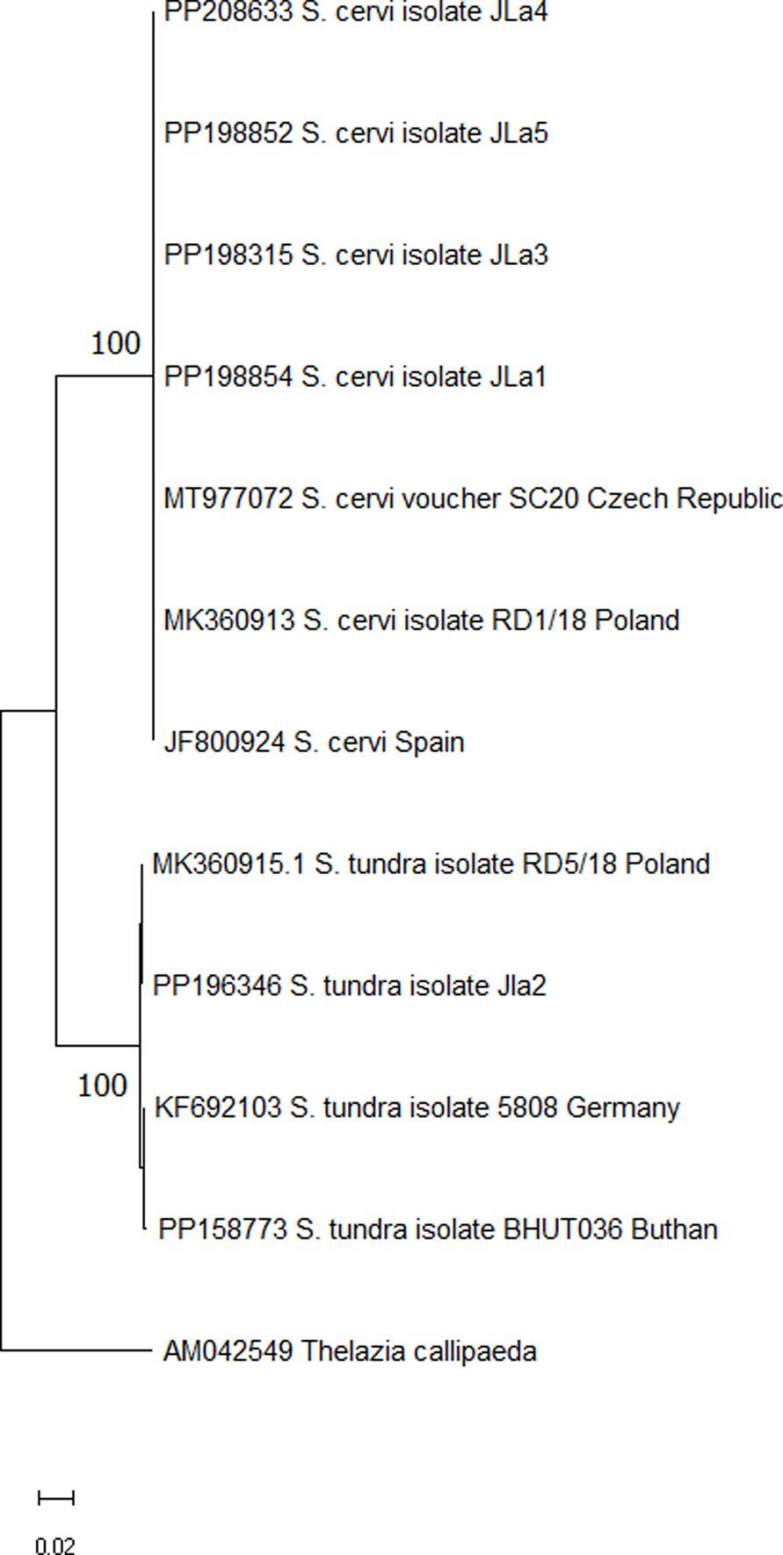
*Setaria* spp. COI phylogenetic tree inferred by the NJ method. The construction of the phylogenetic tree was conducted utilizing the best model Tamura-Nei. Bootstrap analysis was performed with 1.000 replicates; numbers on the tree nodes indicate bootstrap values > 85%. *Thelazia callipaeda* was used as an outgroup. Accession numbers of sequences retrieved from GenBank are indicated together with their geographic origin. Evolutionary analyses were conducted in MEGA 11

To illustrate the genetic relationships between our isolates and the reference isolates, a phylogram was constructed using the Neighbor-Joining (NJ) method (Fig. 4). All isolates of the two *Setaria* species studied were grouped into separate clusters with the highest support values confirming the identification of the species examined. Based on the analysed COI fragment, the intraspecific genetic variation was observed only for *S. tundra*.

## Discussion

Epidemiology of filarial infection especially in red deer has received very little attention. Not many molecular studies have been carried out to describe parasitic infection of *Setaria cervi* in red deer, as far as we know, only a few of them address this topic (Alasaad et al. 2012; Oloś et al. 2019; Lanková et al. 2021). Although in the last century there has been some studies dedicated to morphological descriptions of *S. cervi* such as (Kotrlá et al. 1984; Rajsky 2015).

However, there have been some reports of *S. tundra* occurrence from European countries Germany (Oehm et al. 2022), Denmark (Enemark et al. 2017), Spain (Angelone-Alasaad et al. 2016), Croatia (Čurlík et al. 2019), Austria (Kutzer and Hinaidy 1969), Hungary (Kemenesi et al. 2015), Poland (Tomczuk et al. 2017) and Slovakia (Čurlík et al. 2023), and most importantly there is a recent report of *S. tundra* in red deer (*Cervus elaphus*) in Poland (Oloś et al. 2019). The purpose of our study was to confirm the occurrence of nematode of genus *Setaria* in red deer (*Cervus elaphus*) in Slovakia by using molecular methods. To reach this aim, amplification of the COI gene region was performed, in accordance with similar works (Čurlík et al. 2019, 2023). Consequently, comparison with sequences archived in the GenBank revealed that the analyzed nematodes belong to the species *Setaria tundra* and *Setaria cervi*. Our *Setaria tundra* sequence was compared with sequences in GenBank and revealed the highest sequence similarity (100%-99.42%) with the *S. tundra* sequence (KF692103; PP158773; MK360915) isolated from mosquitoes in Germany, from wolf blood in Australia and in red deer blood in Poland. The final sequences deposited in GenBank showed the highest (100%) sequence similarity to *S. cervi* isolated in Spain from adult nematode and *S. cervi* from red deer in Poland and from *Cervus Nippon* in the Czech Republic (JF800924; MK360913; MT977072).

Infection of *Setaria* is usually harmless, occasionally inducing a mild fibrinous peritonitis which only can be seen at necropsy. Pathological changes may also include dead and partly calcified *Setaria* helminths on the liver surface (Laaksonen and Paulsen 2015). There are no clinical signs when the parasites are in their normal site, although if nervous tissue is involved there is locomotor disturbance which might lead to paraplegia (Taylor et al. 2007), however setariosis with recognizable clinical signs and pathological features can be spotted. The most common findings in reindeer during ante-mortem examination at the slaughterhouses in Finland 2003 were poor body condition, dry fur and underdeveloped winter coat and slightly distended abdomen (Laaksonen et al. 2007). Very typical pathological signs associated with *Setaria* infection during outbreak in Finland 2003 were granulomatous inflammation (greenish or greyish fibrinous membranes covering peritoneum and visceral organs). The surfaces of the livers were typically covered by a thin layer of fibrin and in some severe cases were fibrinous layers presented between all abdominal organs (Laaksonen et al. 2007). Yet, there has not been any report of severe peritonitis associated with *Setaria* infection in roe deer neither red deer. Likewise in this study, no gross lesions of internal organs or any neurological signs associated with *Setaria* infection were observed.

Four out of five positive animals were in good body condition, despite other parasitic infections being presented. We can sum up there was only one individual of red deer (young male/yearling, killed on 04.02.2023) in very poor body condition. Although this particular animal had suffered from multiple parasitic infections such as infection of *Setaria tundra*, gastrointestinal nematodes, larvae of nose botflies, larvae of warble flies and severe *Onchocerca* spp. infection.

All of the *Setaria* helminths were localized in the abdominal cavity which is very typical for this genus (Laaksonen and Paulsen 2015). The prevalence of *Setaria* infection in the present paper was 8.3% which is less in comparison with similar studies conducted in red deer in Poland (72.7%) or Czech Republic (38.5%) (Oloś et al. 2019; Lanková et al. 2021). The intensity of infection was very low, only one specimen of *S. tundra* in positive animal. Compared to the outbreak in Finland 2003 (mean intensity in calves was 8 − 5, with range 0 to 30; mean intensity in adults was 1–5, with range 0 to 84 of *Setaria*) (Laaksonen et al. 2007).

## Conclusion

Infection of *Setaria* spp. has been observed in cervids in several European countries, however this is only the second report of *S. tundra* occurrence in red deer in general and the first report of *S. tundra* and *S. cervi* in red deer in Slovakia. It is only questionable if infections of *S. tundra* and *S. cervi* have started spreading in red deer in recent years or if it has not been objective of any previous studies. The differences of parasitic load of *S. tundra* and *S. cervi* might be caused by unequal biotic and abiotic influence on their life cycle.

## Data Availability

No datasets were generated or analysed during the current study.
